# A profile of a major trauma centre of North West England between 2011 and 2018

**DOI:** 10.1038/s41598-021-84266-x

**Published:** 2021-03-08

**Authors:** Raimundas Lunevicius, Mina Mesri

**Affiliations:** 1 Department of General Surgery, Liverpool University Hospitals NHS Foundation Trust, Cheshire and Mersey Major Trauma Centre Collaborative, University of Liverpool, Aintree Hospital, Lower Lane, Liverpool, L9 7AL UK; 2 North West Schools of Surgery, Health Education England, Regatta Place, Summers Road, Brunswick Business Park, Liverpool, L3 4BL UK

**Keywords:** Diseases, Health care, Medical research

## Abstract

This study examined the trends and patterns of major trauma (MT) activities, causes, mortality and survival at the Aintree Major Trauma Centre (MTC), Liverpool, between 2011 and 2018. The number of trauma team activations (TTAs) rose sharply over time (*n* = 699 in 2013; *n* = 1522 in 2018). The proportion of TTAs that involved MT patients decreased from 75.1% in 2013 to 67.4% in 2018. The leading cause of MT was a fall from less than 2 m (36%). There has been a fivefold increase in the overall number of trauma procedures between 2011 and 2018. Orthopaedic surgeons have performed 80% of operations (*n* = 7732), followed by neurosurgeons, oral and maxillofacial surgeons, and general trauma surgeons. Both types of fall (> 2 m and < 2 m) and road traffic accidents were the three leading causes of death during the study period. The observed mortality rates exceeded that of expected rates in years 2012, 2014, 2016 and 2017. The all-cause observed to expected mortality ratio was 1.08 between 2012 and 2018. A change in care for MT patients was not directly associated with improved survival, although the marginally ascending trend line in survival rates between 2012 and 2018 reflects a gradual positive change.

## Introduction

Between 1990 and 2013, the global rates of injury incidence and associated disability-adjusted life years (DALYs) lost declined by 20% and 31%, respectively^1^. The most significant decreases were seen in the countries of Western Europe, including England, and Australasia^1,2^. Despite the above-mentioned gains, the impact of traumatic injuries on public health remains significant, as major trauma is the leading cause of death in adults under the age of 40 and an important cause of long-term disability in all age groups.

Several systematic deficiencies in the provision of trauma care were identified in England between 2007 and 2010^3,4^. Since then, the public health policy focus has shifted towards the establishment of inclusive regional major trauma networks in the country to improve access to better quality care at the designated trauma centres and reduce trauma-related mortality and the overall burden of injuries^5,6^. In accordance with the Haddon Matrix, the burden of trauma-related injuries is dictated by pre-event, event and post-event factors. In the post-event phase, the provision of a rapid treatment response and rehabilitation for the affected patient may reduce the severity of traumatic disease, and it was believed that a shift towards a more centralised approach was necessary for the efficient provision of such care to those affected by traumatic injury^5,6^.

Currently, there are 27 fully operational Major Trauma Centres for adults and children in England. In the North West region of the country, Liverpool’s Aintree University Hospital was designated as a Major Trauma Centre (MTC) for adult patients in 2012, becoming a central axis for the Cheshire and Mersey Major Trauma Centre Collaborative. This MTC included the Walton Centre for Neurology and Neurosurgery, which is an independent National Health Service Foundation Trust for the region^7,8^. In 2016, Aintree University Hospital became the single MTC covering a population of 2.3 million in the defined sub-region of North West England and the Isle of Man after further centralisation of services for patients with major trauma^9^.

Between 2011 and 2018, Cheshire and Mersey Major Trauma Operational Delivery Network published four versions of standard operating procedure guidelines for local use. The latest version of the guidelines (2017) includes a pre-hospital trauma triage protocol, 'Paramedic Pathfinder', and suggests 23 potential triggers for activation of the major trauma team. They are as follows: three physiological (respiratory rate < 9 or > 30, systolic blood pressure < 110 mm Hg, and GCS < 12), eight anatomical, seven mechanical (by the mechanism of injury), and five various criteria (elderly or frail patient, significant comorbidities, pregnancy, paediatrics, other clinical concern).

Although the benefit of state-wide centralisation of trauma services is well described in the literature^6,8,10,11^, little is known about the change in the epidemiological profile of an individual regional trauma centre of England. The aim of this study was to highlight and assess the trends and patterns of major trauma activities, causes, mortality and survival at a MTC in North West England over the period of 2011 to 2018.

## Methods

This study of retrospective design adheres to the Strengthening the Reporting of Observational Studies in Epidemiology (STROBE) statement and its checklist^12^. It employed a set of data from two sources-Aintree MTC, Liverpool, and Trauma Audit and Research Network (TARN). Firstly, data from Aintree MTC was used to quantify all trauma team activations^12,13^ and surgical procedures performed. Secondly, a subsection of this trauma patient group was defined as having major trauma according to TARN criteria. These included patients whose length of stay in the hospital was 72 h or more, those admitted to a High Dependency Unit regardless of the length of stay and all trauma patient deaths. The number of deaths included those occurring in the Accident and Emergency Department, even if the cause of death was medical. In addition, trauma patients who were transferred from other hospitals for specialist care or utilization of a High Dependency Unit bed with a combined hospital stay at both sites of 72 h or more were included^14,15^.

Based on TARN classification, causes of major trauma were divided into ten categories. They were as follows: fall from less than two metres, fall from more than two metres, road traffic accident (RTA), blow (alleged assault causing blunt trauma), shooting, stabbing, crush, blast, burns and ‘other’. Surgical procedures were performed at Aintree MTC with one exception. Trauma neurosurgical and spinal operations were carried out in the Walton Centre for Neurology and Neurosurgery. Therefore, primary referrals from the region for a neurosurgical procedure at the Walton Centre that bypassed Aintree MTC and Trauma Team were not included in this study. Admissions and deaths from alcohol poisoning and drug overdoses were not included in this study.

Data were entered into standard Microsoft Excel for Mac 2011 spreadsheets and subsequently analysed using standard functions for statistical analysis. We do not report data on trauma team activations for the years 2011 and 2012, as they are incomplete at this preliminary stage. In this study, the percentage change between the numbers of major trauma admissions illustrates the effect size over time. The case-fatality rate was calculated by dividing the number of individuals who died during the period of hospitalization from major trauma by the total number of major trauma patients. The values of observed to expected mortality ratio and the survival rate (Ws) were obtained from the TARN database. These summary measures were calculated using an expected probability of survival values that were derived from a statistical probability of survival model Ps19 using six core characteristics of an injured patient such as age, sex, Injury Severity Score (ISS), Glasgow Coma Score (GCS), pre-existing state of health and a 30-day outcome. It is important to note that where GCS was missing, the category ‘GCS intubation’ was used instead. Furthermore, TARN handled the pre-existing state of health via 21 groups of comorbidities with an allocated weight according to pre-existing data on the strength of the relationship between the specific disease group and clinical outcome. Rate of survival (Ws) was defined as the additional survivors or the reduced number of deaths per year standardised according to the hospital case mix using the TARN fraction^14–16^. A positive rate of survival corresponded to additional survivors per 100 patients as compared to the expected mortality in a defined time. On the contrary, a negative rate of survival corresponded to excess deaths per 100 patients. Relative risk (RR) with 95% confidence interval (CI) for the binomial proportions from case-fatality rates were computed using Koopman asymptotic score method via GraphPad Prism Version 8.3.1 (332) for MacOS (GraphPad Software, Inc., La Jolla, CA, USA). The rates of observed and expected mortality with 95% CIs for each year were obtained from the TARN as they have been calculated using TARN-specific probability of survival Ps19 model.

Research registration and ethical approval from the research ethics committee was not required as confirmed by the National Research Ethics Service decision tool^17^. However, the institutional clinical audit management board reviewed, approved and registered the submitted protocol of the study as a no-risk audit (No. 6977). Acquisition of informed consent from patients was not required. The authors had no access to identifying patient information when collecting and analysing the data. We confirm that the study does not include identifiable information about individual patients.

## Results

### Trauma team activations and major trauma admissions

**Figure 1:**
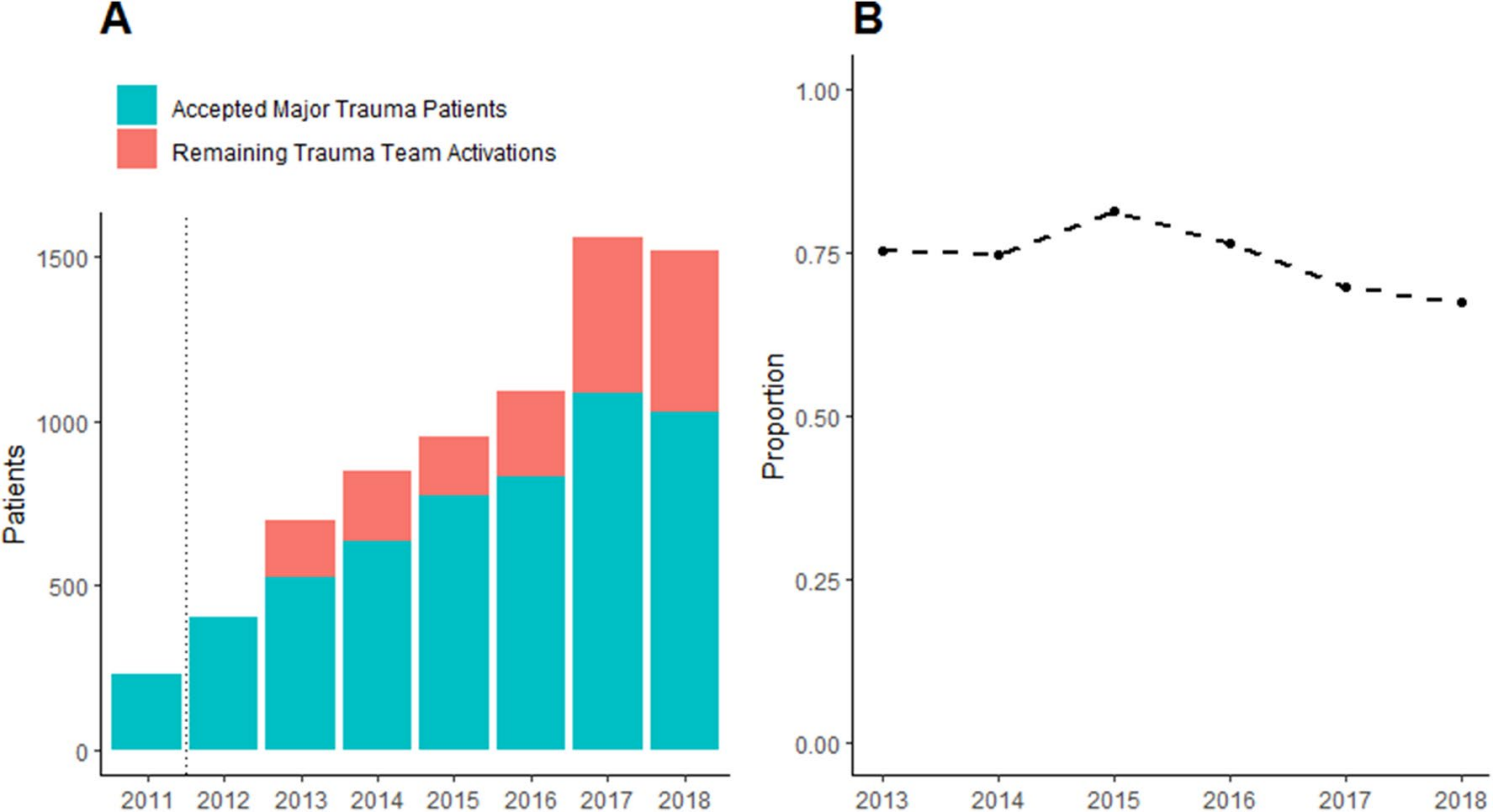
Trends and patterns from trauma team activations and major trauma patients at Aintree Major Trauma Centre, Liverpool, between 2013 and 2018. (**A**) Number of trauma team activations and accepted major trauma patients. The number of trauma team activations for the years 2011 and 2012 was not included, as complete data were not available for these years. (**B**) The annual proportion of trauma team activations that were accepted as major trauma patients at Aintree Major Trauma Centre, 2013 to 2018.

The total number of accepted major trauma patients at Aintree hospital increased from 230 in 2011 to 1,025 in 2018 in the years following its establishment as a MTC. While a record of activations in 2011 and 2012 was not available, the number of trauma team activations increased from 699 in 2013 to 1,522 in 2018 (Fig. 1A). Overall, between 2013 and 2018, there were 6975 trauma team activations, of which 69.9% (n = 4875) constituted confirmed major trauma patients. However, the proportion of trauma team activations that were accepted as major trauma patients decreased over this time period, from 75.1% in 2011 to 67.4% in 2018 (Fig. 1B).

### Main causes of traumata

The five most common causes of traumatic injury were falls from less than two meters (36%, n = 1958 out of 5,509), road traffic accidents (25%, n = 1388), falls from more than two meters (22%, n = 1220), blunt trauma secondary to alleged assault (10%, n = 557) and stabbings (4%, n = 202). Falls and road traffic accidents, the most common causes of blunt traumatic injuries, collectively accounted for 83% (n = 4566) of admissions over eight years.

**Figure 2:**
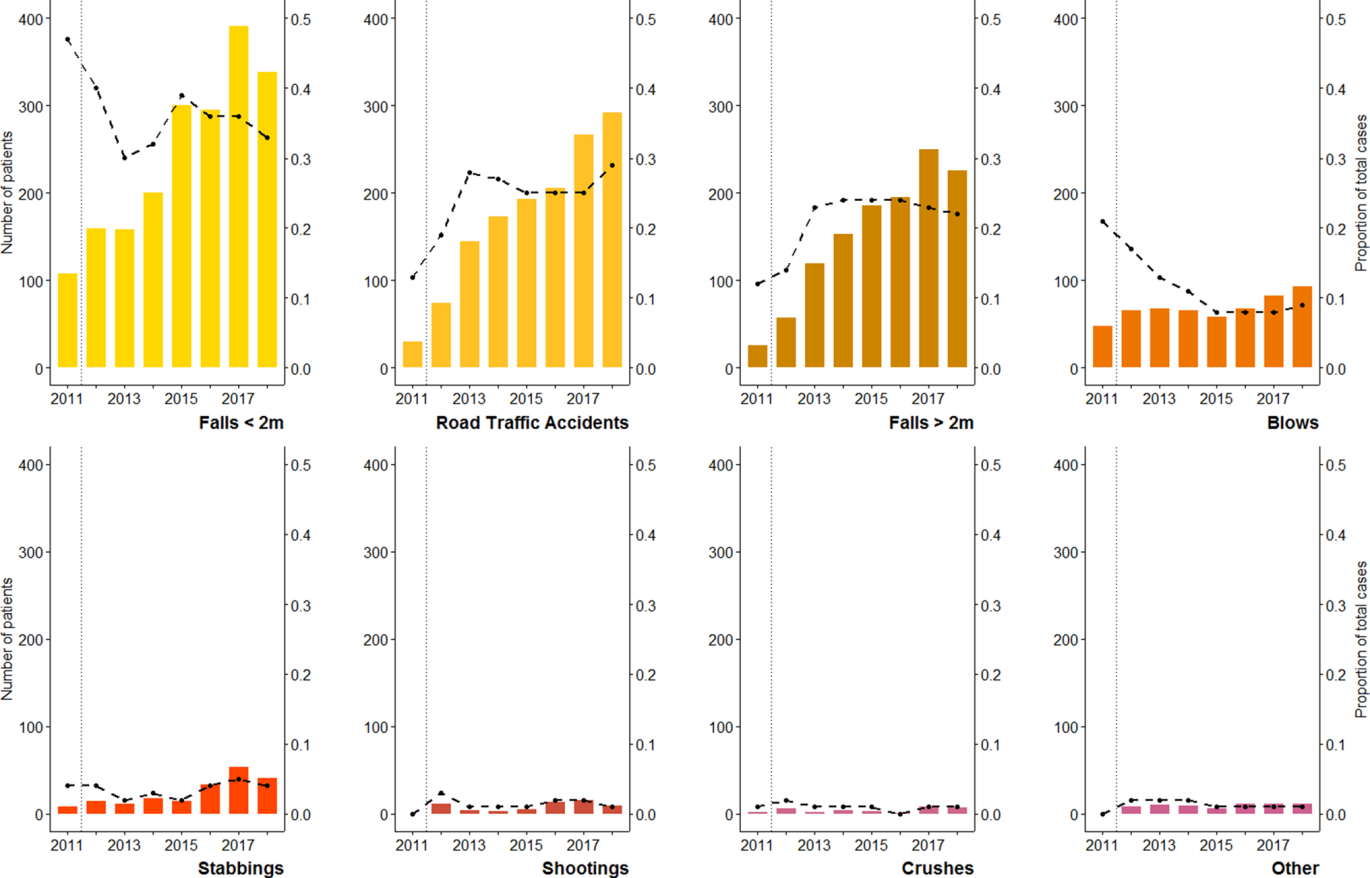
The number of patients admitted following each of eight leading causes of major trauma to Aintree Major Trauma Centre, Liverpool, from 2011 to 2018. The vertical dotted lines indicate the year 2012, in which Aintree became an established Major Trauma Centre. For each cause of admission (burns and blasts not shown as they were sporadic) the proportional contribution to the total number of major trauma patients is indicated by the black dashed line. Blows = alleged assaults, causing blunt traumata.

Mirroring the overall increase in the number of major trauma patients, each cause of injury typically saw increasing patient numbers between 2011 and 2018 (Fig. 2). However, there were notable changes in their proportional contribution to the total number of major trauma patients seen at Aintree Hospital in the years immediately following its establishment as a MTC. Falls from less than two metres, the most common cause of trauma, made up 47% of all major trauma patients in 2011, but by 2013 this had reduced to 30%. Similarly, the proportion of patients presenting with blunt trauma secondary to alleged assault (blows) decreased from 21% in 2011 to 13% in 2013. On the contrary, the proportion of patients presenting as a result of a fall of greater than two metres rose from 12% in 2011 to 23% in 2013, while the proportion of patients presenting following a road traffic accident increased from 13% in 2011 to 28% in 2013. This dramatic change in the overall patient profile appeared to plateau after this point, and little variation was seen between 2013 and 2018, while the proportion of stabbings, shootings, crushes and other injuries remained relatively stable throughout the study period.

### Surgical procedures

In total, 7,732 surgical procedures were performed between 2011 and 2018, with trauma and orthopaedic surgeons performing the vast majority of these (80.1%, n = 6195). The remaining operations were performed by neurosurgeons (5.9%, n = 435), while oral and maxillofacial surgeons contributed to 4.6% (n = 359) and general surgeons to 2.2% (n = 169; five of which were laparoscopic procedures). Thirty-three resuscitative thoracotomies (0.43%) and 162 vascular procedures (2.1%) were performed. In each case, a primary procedure to gain access to the vasculature such as thoracotomy, laparotomy, minimal access procedures (interventional radiology) or targeted incisions for classified arteries or veins was performed beforehand.

**Figure 3:**
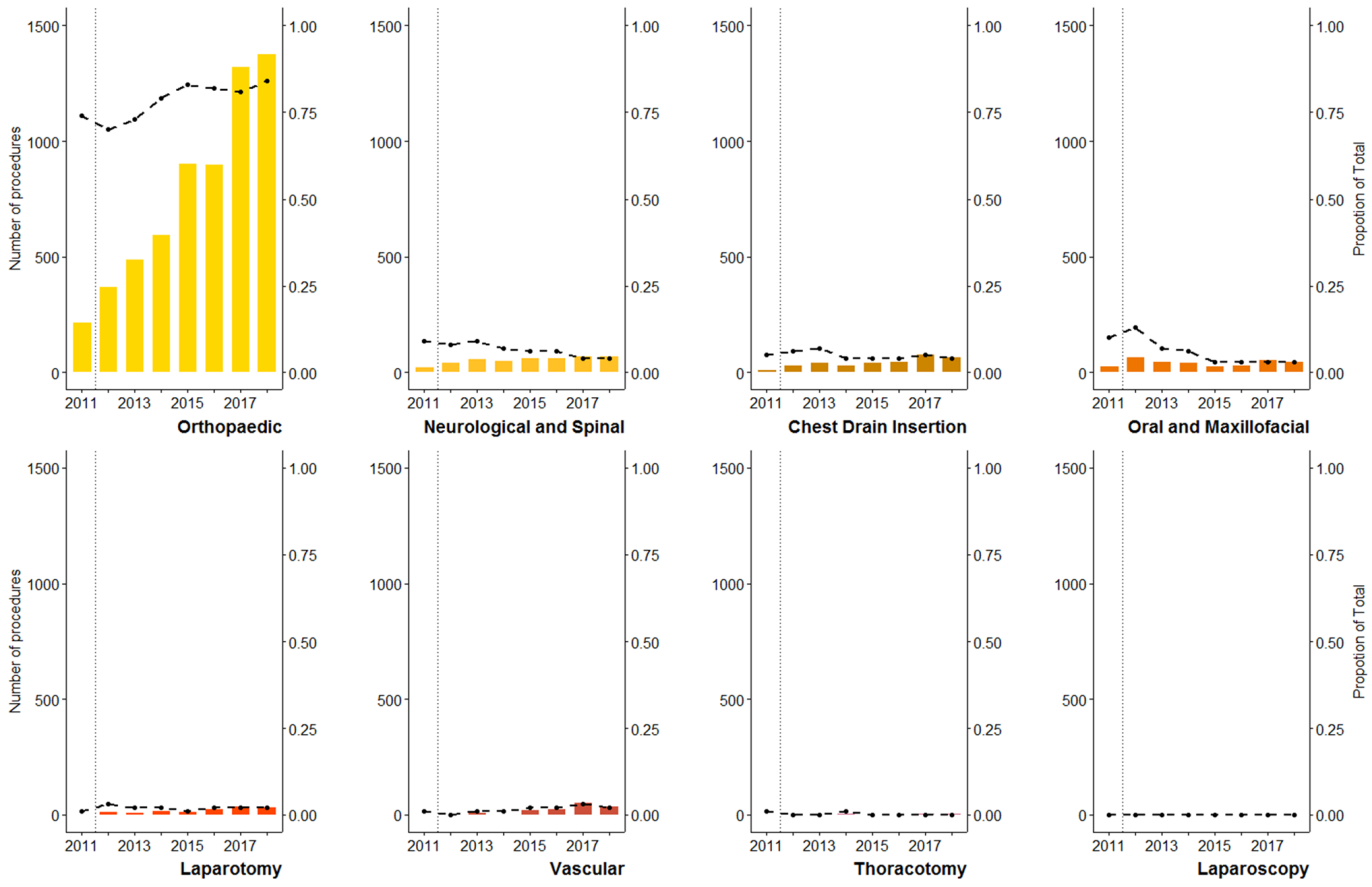
The frequency of surgical procedures performed by each surgical sub-speciality for major trauma patients at Aintree Major Trauma Centre, Liverpool, 2011 to 2018. The vertical dotted lines indicate the year 2012, in which Aintree became an established Major Trauma Centre. For each sub-speciality, the proportional contribution to the total number of procedures performed on major trauma patients is indicated by the black dashed line. 298 surgical procedures performed in 2011; 536 in 2012; 667 in 2013; 758 in 2014; 1,087 in 2015; 1,104 in 2016; 1,636 in 2017; 1,646 in 2018.

As demonstrated by Fig. 3, the total number of procedures performed by trauma and orthopaedic surgeons increased from 220 in 2011 to 1,381 in 2018. As a proportion of the total surgical procedures received by major trauma patients, the number performed by trauma and orthopaedic surgeons increased from 73.8% in 2011 to 83.9% in 2018. This coincided with a relative reduction in the proportion of neurological and spinal procedures, from 8.7 to 4.3%, and in oral & maxillofacial surgeries, from 10.1 to 2.9%, while the proportion of all other procedures remained relatively stable throughout the study period.

### Case fatalities

**Figure 4:**
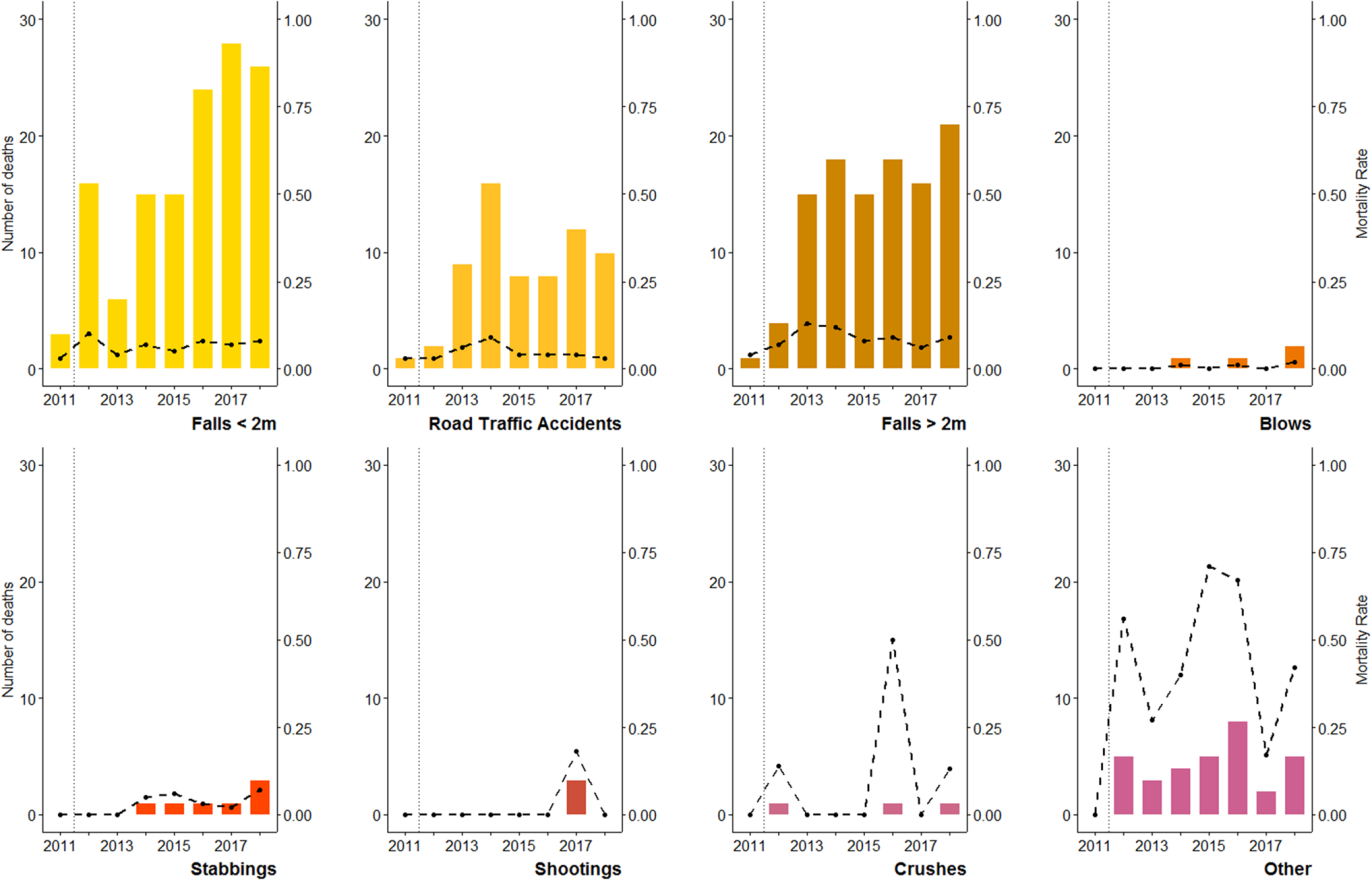
The number of patient deaths following admission from each of eight leading causes of major trauma at Aintree Major Trauma Centre, Liverpool, 2011 to 2018. The vertical dotted lines indicate the year 2012, in which Aintree became an established Major Trauma Centre. For each cause of admission, the black dashed line indicates the annual case-fatality rate.

Figure 4 shows the annual number of deaths between 2011 and 2018, by cause of major trauma. In 2011, there were five deaths following major trauma admission at Aintree Hospital. In 2012, following its establishment as a MTC, this number increased to 28, and a gradual increase was seen over the following years. In 2018, there were 68 deaths from major trauma, an increase of 143% compared to the number seen in the MTC’s first year. In total, there were 356 deaths as a result of major trauma between 2011 and 2018. Falls from less than two meters (133 deaths, 37% of all deaths), falls from more than two meters (108 deaths, 30%), and road traffic accident (66 deaths, 19%) were the leading causes of death from major trauma between 2011 and 2018. In total, 86.2% of all deaths (307 of 356) were attributed to falls and road traffic accidents. The proportional contribution to the total number of deaths by each of these three causes remained relatively stable during the study period.

The case-fatality rate was 6.5% for all-cause major trauma admissions between 2011 and 2018. It significantly increased in the first year Aintree MTC was established, from 2.2% in 2011 to 6.9% in 2012 (RR 3.18, 95% CI 1.25–8.14). However, the all-cause trauma case-fatality rate remained relatively stable from 2012 to 2018 (6.6%; RR 0.96, 95% CI 0.63–1.46). The case-fatality rate was found to be highest in the group of patients admitted under the cause-of-injury category ‘other’ (39.9%). The case-fatality rate from falls under two metres (6.8%; range 2.8–10%) was similar to those from falls greater than two metres (8.9%; range 3.7–12.5%), while the case-fatality rate from RTA injuries was 4.8% (range 2.8–9.2%). No substantial variation was seen in the case-fatality rate for any of the individual causes of trauma between 2011 and 2018.

### Observed and expected mortality rates

**Figure 5:**
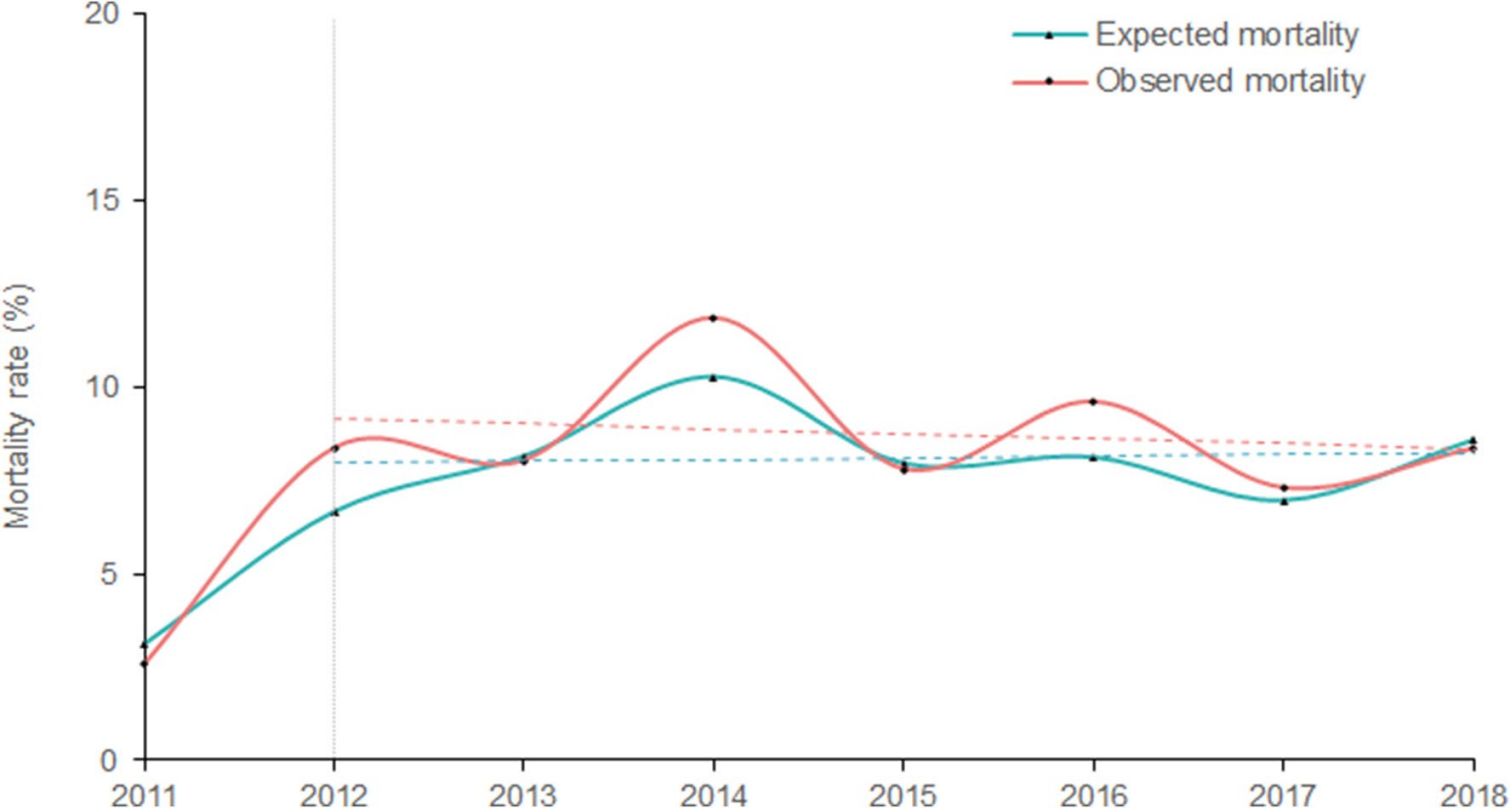
The observed and expected all-cause mortality rates at Aintree Major Trauma Centre, Liverpool, 2011 to 2018. The vertical dotted lines indicate the year 2012, in which Aintree became an established Major Trauma Centre. The dashed lines represent the linear trends for observed and expected all-cause mortality following the Major Trauma Centre's establishment. The rates of observed (crude) and expected (predicted) mortality with 95% CIs are as follows: 8.36% (7.89 to 8.83) and 6.67% (6.30 to 7.05) for the year 2012; 8.03% (7.66 to 8.40) and 8.15% (7.78 to 8.52) for 2013; 11.83% (11.36 to 12.30) and 10.25% (9.84 to 10.66) for 2014; 7.80% (7.54 to 8.06) and 7.96% (7.70 and 8.23) for 2015; 9.59% (9.31 to 9.87) and 8.12 (7.88 to 8.36) for 2016; 7.30% (7.14 and 7.47) and 6.97% (6.81 to 7.12) for 2017; 8.35% (8.16 to 8.55) and 8.58% (8.38 to 8.78) for the year 2018.

Figure 5 demonstrates the observed and expected mortality rates by year. They fluctuated between 2011 and 2018. The observed mortality rate was measured at its lowest in 2011, at 2.6%. The observed mortality rates exceeded that of expected rates in years 2012 (8.36% vs. 6.67%), 2014 (11.83 vs. 10.25%), 2016 (9.59% vs. 8.12%) and 2017 (7.30% vs. 6.97%). Despite these fluctuations, the overall rate of observed mortality appears to decline slightly between 2012 and 2018.

### Rate of survival

**Figure 6:**
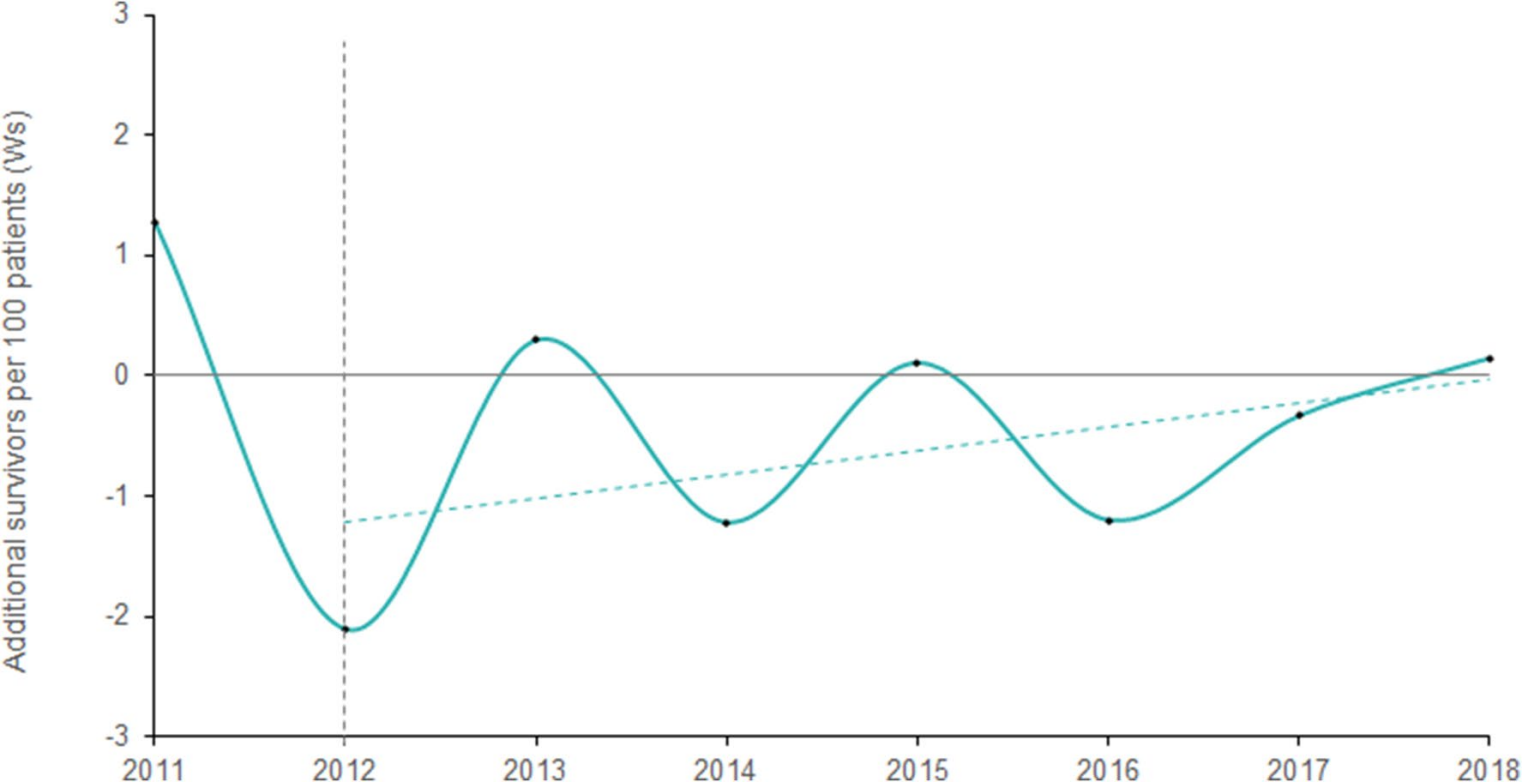
Estimated survival rates (Ws)-the additional survivors per 100 patients admitted at Aintree Major Trauma Centre compared to the expected mortality. The vertical dotted line indicates the year 2012, in which Aintree became an established Major Trauma Centre. The dashed line represents the linear trends for Ws following the establishment as a Major Trauma Centre. The estimated rates of survival with 95% CIs are as follows: 1.28 (− 3.53 to 6.08) in 2011, − 2.11 (− 4.71 to 0.49) in 2012, 0.3 (− 1.98 to 2.58) in 2013, − 1.22 (− 3.22 to 0.79) in 2014, 0.11 (− 1.72 to 1.94) in 2015, − 1.2 (− 2.98 to 0.58) in 2016, − 0.33 (− 1.82 to 1.15) in 2017, and 0.15 (− 1.37 to 1.67).

Figure 6 demonstrates the additional survivors per 100 patients. At 1.28 additional survivors per 100 patients (95% CI − 3.53 to 6.08), the highest rate of survival was observed in 2011 (pre-MTC era). Overall, during the period of 2011–2018, the estimations were found to be highly fluctuant. Excess deaths were observed in 2012, 2014, 2016 and 2017, and additional survivors in very modest numbers in 2013 (0.3 within 95% CI − 1.98 to 2.58), 2015 (0.11 within 95% CI − 1.72 to 1.94), and 2018 (0.15 within 95% CI − 1.37 to 1.67). However, the overall trend-line from the mean rates of additional survivors between 2012 and 2018 was found to be ascending.

## Discussion

The fundamental goal of a trauma system is to save and prolong life while limiting the morbidity caused secondary to traumatic injury. A trauma centre is an essential component of a trauma system where the resources and expertise are available to provide the required initial resuscitation, definitive trauma surgery and early rehabilitation. This paper focuses on the general analysis of the key trauma activities, causes of injury, in-hospital mortality and survival in a MTC for Cheshire and Mersey region of North West England between 2011 and 2018.

Following the launch of the Major Trauma Centre in 2012, we detected significant increases in major trauma team activations, admissions to the hospital, observed all-cause mortality rate and the ratio of observed to expected all-cause mortality rates. In the following years, the ratio of observed to expected all-cause mortality rates gradually declined while the survival rate slowly increased. Six other themes emerge from the findings of this study.

First, the substantial increase of major trauma team activations from 699 to 1,522 between 2013 and 2018 is secondary to the increasing centralisation of trauma care in England^7,8^. A further rise in the number of patients admitted with major trauma would exhaust the MTC’s resources and have significant implications on other essential functions of an acute care system. However, given the plateau observed in 2017–2018, we propose that such an increase in major trauma team activations is unlikely, particularly as the incidence and prevalence of major trauma changes slowly over time^1^. However, effective coordination between the pre-hospital emergency care providers, major trauma consultants and professionals from local hospitals are the supplemental ways to control and divert the flow of patients with suspected mild-to-moderate injuries to these hospitals.

Second, it is not clear why the significant increase of major trauma team activations observed during the study period resulted in a 8% reduction in fractions of major trauma patients (from 75 to 67%). This change represents an increase in inappropriate trauma team activations between 2013 and 2018. We presume that the ineffective communication between the pre-hospital and hospital care providers, and the dogmatic interpretation of the suggested anatomical, physiological, mechanical and other triggers for major trauma team activations are the root causes of inappropriate major trauma team activations^13,18^. Our findings suggest the need for a re-evaluation of pre-hospital trauma triage criteria and trauma team activation protocols at our major trauma centre^18,19^.

Third, 95% of patients were admitted with injuries caused by falls and road traffic accidents. This is not a surprising finding, as similar patterns have been found in other trauma centres globally^20^. However, the large proportion of patients (83%) admitted with significant injuries following a fall or road traffic accident and high cause-specific case-fatality rates (up to 10–12.5% in 2012 and 2013) are alarming findings that underline the importance of preventative measures and programmes on road safety and falls; known as a systematic work for trauma pre-event phase^5,21^.

Fourth, a worrisome 420% increase in penetrating injuries from stabbing and shooting (from 10 in 2011 to 52 in 2018) is a dangerous trend in interpersonal violence and self-harm in North West England's geographical region. It has recently been stressed that over one-quarter of young violent trauma patients will be readmitted to a MTC with a second unrelated trauma or will die within five years after primary discharge^22^.

Fifth, the centralisation of trauma services led to a sudden change in the proportions of major trauma admissions by cause-of-injury. These proportions remained relatively stable throughout the following six years.

Sixth, excess deaths were observed in the years 2012, 2014, 2016 and 2017. However, the ascending trend-line in survival rates between 2012 and 2018 reflects a gradual positive systematic change in pre-hospital and hospital medical infrastructure, the concentration of experience, trauma-specific knowledge, innovations and multidisciplinary approach contributed to an improvement in the gradual trauma care quality and the reduction in case-fatality rates on an all-cause scale of trauma, especially from 2015 to 2018.

One limitation of this study is that additional data on 356 trauma-related mortalities between 2011 and 2018 were not collected. The absence of data on basic patient demographics, pre-existing medical history, injury diagnosis and immediate cause of death restricted us from assessing the patterns of trauma deaths by age, sex, Charlson comorbidity index, and nature-of-injury, preventing us from drawing broader conclusions^1,23,24^. However, this study shows that trauma orthopaedic surgery and neurosurgery are vital on-site specialities required to provide damage control and definitive surgical intervention for trauma patients^25,26^. It is important to note that the overall proportion of trauma neurosurgical procedures is much higher, as a substantial number of patients with isolated injuries to the brain or spine requiring surgery are directly transferred to the Walton Center for Neurology and Neurosurgery from other health care facilities of the region, after direct consultation with a neurosurgeon on call bypassing Aintree MTC. A further limitation of this study arose from the shortcomings associated with its conception. For example, self-inflicted injuries were not estimated separately for this analysis.

This study demonstrates that improved quality of trauma care in a new MTC is not a straightforward linear process^27,28^. The key message is that the measured annual observed and expected mortality and survival rates varied considerably between 2011 and 2018, with an unexpected surge in mortality observed in 2012 immediately following the launch of the Major Trauma Centre Collaborative in Merseyside and Cheshire, North West England. Although we found declining mortality rates, we presume that the region's population would benefit from revised or new local trauma policies and quality improvement programmes to consistently achieve low observed to expected mortality ratios over the next seven years.

In conclusion, Aintree MTC’s profile has significantly changed since its establishment in 2012. A change in the organisation of care for patients with severe traumatic injuries was not directly associated with substantial improvement in survival rates, although the marginally ascending trend line in survival rates between 2012 and 2018 reflects a gradual positive change. An annual analysis of the trends, patterns and potential contributing factors for trauma mortality is required to improve trauma work, encouragement and accountability within the institution to achieve higher survival rates for major trauma patient cohorts. This report should aid those responsible for devising regional trauma system and major trauma centre collaborative policy to re-formulate strategies that ensure optimal trauma care for the region's population.

## Data Availability

Data generated and analysed during this study are included in this article. Study-specific Microsoft Excel for Mac 2011 files are available from the corresponding author on reasonable request.
